# Efficacy of ketogenic metabolic therapy as an adjuvant to the current standard of care in the treatment of glioblastoma: A systematic review of clinical trials

**DOI:** 10.1007/s12032-025-03165-7

**Published:** 2025-12-11

**Authors:** Emily McKerill, Joecelyn Kirani Tan, Chethana Krishna Rao, Christian A. Linares, Soirindhri Banerjee, Rukhshana Dina Rabbani, Sola Adeleke, Aruni Ghose, Stergios Boussios

**Affiliations:** 1 School of Medicine, Faculty of Medicine and Health, University of Leeds, Leeds, UK; 2 Leeds Oncology Society, Leeds, UK; 3 School of Medical Sciences, Faculty of Biology, Medicine and Health, University of Manchester, Manchester, UK; 4 Department of Clinical Oncology, The Christie NHS Foundation Trust, Manchester, UK; 5 British Oncology Network for Undergraduate Societies, Liverpool, UK; 6 Barts and The London School of Medicine and Dentistry, Queen Mary University of London, London, UK; 7 Department of Oncology, Havering and Redbridge University Hospitals NHS Trust, Barking, London, UK; 8 Department of Cancer Imaging, School of Biomedical Engineering & Imaging Sciences, Faculty of Life Sciences and Medicine, King’s College London, London, UK; 9 Cancer Centre at Guy’s, Guy’s and St Thomas’ NHS Foundation Trust, London, UK; 10 Department of Clinical Oncology, The Royal Marsden NHS Foundation Trust, London, UK; 11 Barts Cancer Centre, St Bartholomew’s Hospital, Barts Health NHS Trust, London, UK; 12 Barts Cancer Institute, Queen Mary University of London, London, UK; 13 Department of Research and Innovation, Medway NHS Foundation Trust, Gillingham, UK; 14 Health Systems and Treatment Optimisation Network, European Cancer Organisation, Brussels, Belgium; 15 Department of Medical Oncology, Ioannina University Hospital, Ioannina, Greece; 16 Faculty of Medicine, School of Health Sciences, University of Ioannina, Ioannina, Greece; 17 AELIA Organization, 9th Km Thessaloniki-Thermi, 57001 Thessaloniki, Greece; 18 Faculty of Medicine, Health and Social Care, Canterbury Christ Church University, Canterbury, UK; 19 School of Cancer and Pharmaceutical Sciences, Faculty of Life Sciences and Medicine, King’s College London, London, UK; 20 Kent and Medway Medical School, University of Kent, Canterbury, UK

**Keywords:** Ketogenic metabolic therapy, Efficacy, Adjuvant, Glioblastoma, Brain tumour

## Abstract

Glioblastoma is a diffuse, heterogenous tumour with a poor prognosis as current therapeutic options have limited efficacy. As a result, research aims to explore new treatment options which exploit the hallmarks of cancer. This review aimed to understand the breadth of research considering ketogenic metabolic therapy (KMT) as an adjuvant to standard therapy. KMT aims to improve overall survival by exploiting the metabolic reprogramming exclusive to neoplastic cells. Preclinical trials show benefits in KMT when used alongside radiotherapy, through increasing anti-tumour effects compared to controls. Literature searches conducted over three databases, in line with PRISMA guidelines, collated studies relevant to KMT and glioblastoma. Six prospective studies and one retrospective study met the inclusion criteria for this review. Data regarding participants, interventions and survival were extracted. Studies included used small numbers of participants, as many aimed to assess the feasibility of larger-scale trials, which increases errors and bias of results. Furthermore, direct comparison between trials was limited due to study heterogeneity, as each trial used differing parameters and diet compositions. As a result, no definitive conclusions could be made. Future studies should use larger cohorts with standardised parameters so results are representative, and comparisons can be made to evaluate efficacy.

## Introduction

The World Health Organisation classifies glioblastoma multiforme as a grade 4 tumour of the central nervous system [1], indicating a poor prognosis. Glioblastoma mortality rate is influenced by the limited treatment options. There is a median survival of 15 months when treated aggressively with surgical resection, radiotherapy and chemotherapy compared to three months with surgery alone [2]. No link has been found between glioblastoma and other known carcinogens, except for ionising radiation therapy, although the latency period is unknown [3]. However, glioblastoma prevalence increases with age, as the mean age of diagnosis is 62 years [4].

Clinical presentation varies depending on tumour location [5]. Tumours in the frontal lobe can affect a patient’s personality, whereas tumours in the occipital lobe can result in vision loss. Seizures are experienced by more than 60% of glioblastoma patients, with many going on to develop brain tumour-related epilepsy. Diagnosis is difficult as many symptoms are non-specific, including headaches, fatigue and cognitive decline. Following the presentation, immediate imaging is required. Magnetic resonance imaging (MRI), if not contraindicated, is used as MRI is more sensitive to glioblastoma than computed tomography [6]. Specific MRI scans are used to understand tumour features, including T1-weighted gadolinium enhancement, fluid-attenuated inversion recovery and MR spectroscopy [7]. Despite extensive imaging methods, a definitive diagnosis of glioblastoma occurs through histological analysis. Major histological features include microvascular proliferation and necrosis, both central due to insufficient blood supply and irregular necrotic foci spread throughout the tumour [8]. Other features include nuclear hyperchromatism, an increased mitotic index and anaplasia.

The first line of treatment, currently, is maximal surgical resection. However, due to the infiltrative nature of the tumour, complete resection is virtually impossible without causing major deficits to the patient [9]. Introducing 5-aminolevulinic acid hydrochloride (5-ALA) to aid the visualisation of tumour margins enhanced the extent of safe surgical resection [10]. Retrospective studies have shown the use of 5-ALA within surgery has increased the 6-month progression-free survival rate (PFS) by increasing gross resection [11]. In addition to surgery, both radiotherapy and an alkylating chemotherapeutic agent, temozolomide (TMZ), are considered standard therapy. A randomised trial by Stupp et al. (2005) [12] showed the benefits of concomitant TMZ therapy with a median increase of 2.5 months in survival when compared with radiotherapy alone. Following TMZ treatment, there is a high immunosuppression risk, thus trimethoprim-sulfamethoxazole prophylaxis is prescribed to prevent opportunistic infections [13]. Symptomatic relief can be given through the prescription of antiepileptics, such as levetiracetam, for seizures [14]. Dexamethasone, a corticosteroid, is used to reduce radiotherapy-related oedema; following improvement, this dosage is titrated down to prevent side effect accumulation [11].

Prognosis for glioblastoma remains low, even when treated with current options, with most patients surviving between 12 and 18 months from initial diagnosis [5]. Treatment resistance occurs through various mechanisms. Mutations causing glioblastoma result in the alteration and suppression of genes causing oncogenesis in three distinct cell lineages producing a heterogenic tumour [15]. This heterogeneity contributes to the inefficacy of current treatments. Moreover, glioblastoma is highly infiltrative producing microscopic projections into other brain regions, providing difficult margins for surgical resection [9]. Hypotheses also suggest neoplastic cells in the tumour periphery can quiesce, reducing the likelihood of resection and increasing the risk of recurrence [16]. The presence of the blood-brain barrier limits therapeutic options as specific drugs are not permeable and cannot reach the target area [9].

Mutations influence prognosis and treatment efficacy. Methylation of the O^6^-methylguanine-DNA methyltransferase (MGMT) promoter region determines TMZ effectiveness. This gene codes for a ubiquitously expressed DNA repair enzyme which reverses alkylation damage caused by TMZ. Where TMZ alkylates the O^6^ site on guanine, MGMT removes this adduct, preventing damage and thus preventing cell death [17]. Methylation of this promoter region silences MGMT causing glioblastoma cells to be vulnerable to TMZ. This correlates to a favourable survival in glioblastoma patients, with a median overall survival (OS) of 21.7 months when methylated and 12.7 months when not. However, the mutation is only prevalent in 35% of glioblastoma patients [6]. Moreover, mutations in the isocitrate dehydrogenase enzymes (IDH) convey a favourable outcome compared to wild-type tumours. Observations show an OS of 31 months in IDH-mutated tumours compared to 15 months in IDH wild-type patients [18]. Although this study showed that younger patients were diagnosed with IDH-mutated tumours, there was a median age of 32 years for those with the mutation and 59 years for those without, which could influence OS.

Emerging treatments for glioblastoma include immunotherapy and targeted therapy. Randomised controlled phase II trials studying the use of dendritic cell vaccines containing synthetic peptides targeting glioblastoma-specific antigens have shown benefits in PFS compared to controls [19]. However, phase III trials are required before approval. Stupp et al. (2017) [20] considered tumour-treating fields (TTF) as an adjuvant to TMZ in glioblastoma patients. This trial showed an increase in OS with no limits to quality of life when TTF was paired with TMZ in comparison to TMZ alone. This contrasts with a study by Seyfried et al. (2011) [21], considering the metabolic management of glioblastoma by implementing ketogenic metabolic therapy (KMT) due to the adaptations in glucose metabolism in glioblastoma cells. Case reports also support this, showing a reduction in the tumour size two months post-treatment [22].

KMT, regardless of composition, consists of a high-fat, low-carbohydrate diet [23]. Many studies use 3:1 or 4:1, fat: carbohydrate, ratios, causing cells to metabolise ketones over glucose for energy. Ketogenic diets (KD) mimic the metabolic fasting state by reducing blood glucose [24]. Clinical benefits include anticonvulsant, anti-inflammatory, and antioxidative effects, with KD recommended to treat epilepsy [25], diabetes [26], and cancer [27].

Normal cells metabolise energy through aerobic glycolysis and oxidative phosphorylation. Metabolic reprogramming in tumour cells leads to an emphasis on aerobic glycolysis, reducing oxidative phosphorylation, when metabolising energy; this is deemed the Warburg effect [28]. Jelluma et al. (2006) [29] showed the high demand for glucose in glioma cells via glucose withdrawal. Cell death occurred as aerobic glycolysis could not occur leading to oxidative stress through the overproduction of mitochondrial oxygen free radicals, causing apoptosis. This did not occur in the control cells, normal human astrocytes. Aerobic glycolysis is less efficient at producing ATP, thus the demand for glucose is higher in glioma cells [24]. This can be exploited therapeutically. Reducing the blood glucose concentration through KD results in a shift in energy metabolism from using glucose as a substrate to ketone bodies [30], which yields more energy via oxidative phosphorylation than glucose, and thus is more efficient [31]. However, in cancer cells, metabolic reprogramming emphasises ATP production through glycolysis, restricting the ability to use ketone bodies. Reducing glucose levels via KD induces metabolic stress as tumour cells are maladapted to using ketone bodies for energy metabolism. This reduces the availability for nucleotide and ATP synthesis, forcing cells into a pro-apoptotic state and restricting tumour growth [28].

Abdelwahab et al. (2012) [32] studied KMT with radiotherapy in implanted glioblastoma tumours in mice. Results showed a statistically significant difference in the prolonged survival of the mice, with those fed a standard diet (SD) surviving a median of 23 days compared to those on a 6:1 KD diet surviving 28. Moreover, KetoCal^®^, a KD, was found to cure the tumours. For those treated with radiation and KetoCal^®^, there was an exponential decline in the bioluminescent signal, used to detect glioma cells, from day nine. This remained undetectable until day 104 when the mice were converted to an SD, where there was no detectable recurrence found before study completion [32]. Furthermore, KMT has proven to be beneficial for other cancers. Jemal et al. (2021) [33] completed a systematic review showing trials studying KMT and breast cancer. KMT was found to increase the response to therapeutic drugs. However, results stated more complex, randomised controlled trials were required to confirm this, despite most preclinical data supporting KMT [33].

Previous systematic reviews have considered KMT as a treatment option for glioblastoma. Pangal et al. (2021) [34] studied complementary and alternative medicine when treating gliomas including KMT as well as hyperbaric oxygen and antioxidants. Whereas Martin-McGill et al. (2018) [35] solely focussed on the role of KMT but considered this a treatment option for both adult and paediatric gliomas rather than glioblastoma specifically. This systematic review aims to understand the scope of evidence detailing the use of KMT as an adjuvant to radiotherapy and chemotherapy in the treatment of glioblastoma. The eligibility criteria used ensured the efficacy of KMT alone was studied rather than incorporating other supporting therapies to understand if this is a feasible and clinically beneficial option for patients diagnosed with glioblastoma.

## Methods

This study aims to review the efficacy of KMT as an adjuvant to the current standard of care in patients with glioblastomas by evaluating the effect of KMT on overall survival and progression-free survival.

Following the criteria outlined by PRISMA (Preferred Reporting Items for Systematic Reviews and Metanalyses), a literature search of three databases was completed surrounding the use of KMT in the treatment of glioblastoma [36]. The systematic search was completed on PubMed, Scopus, and Medline (Ovid) using seven possible strings, reviewing all available search fields, including titles, abstracts, keywords and subject headings. The strings included: Glioblastoma and Ketogenic Metabolic; Glioblastoma Multiforme and Ketogenic Metabolic; Glioblastoma Multiforme and Ketogenic; Glioblastoma and Ketogenic; Glioblastoma and KMT; Glioblastoma and Ketogenic Metabolic; Glioblastoma and KMT.

Completing the data retrieval process on two separate occasions, 15th February 2023 and the subsequent search on 22nd March 2023 ensured all relevant articles were included and no additional publications had been made. Studies were collated into an EndNote library (version 20), used to remove all duplicates. The collated research spanned the last 16 years, including all reviews and initial research into KMT. The papers included were subject to extensive filtering, including the removal of any gray literature, primary prevention studies, and reports. Subsequent filtering, including analysis of the abstracts and the full papers, resulted in a total of 25 papers identified as relevant to this review. Further screening using the inclusion and exclusion criteria, Table 1, eliminated 17 papers which did not focus on glioblastoma and included both KMT and standard therapy. Studies were excluded if there were additional non-conventional therapies used alongside KMT, such as hyperbaric oxygen therapy [37] or the administration of intranasal perillyl alcohol [38]. Following this screening, a total of seven relevant papers were found to analyse clinical trials which studied KMT as an adjuvant to the current standard of care for glioblastoma.

**Table 1:**
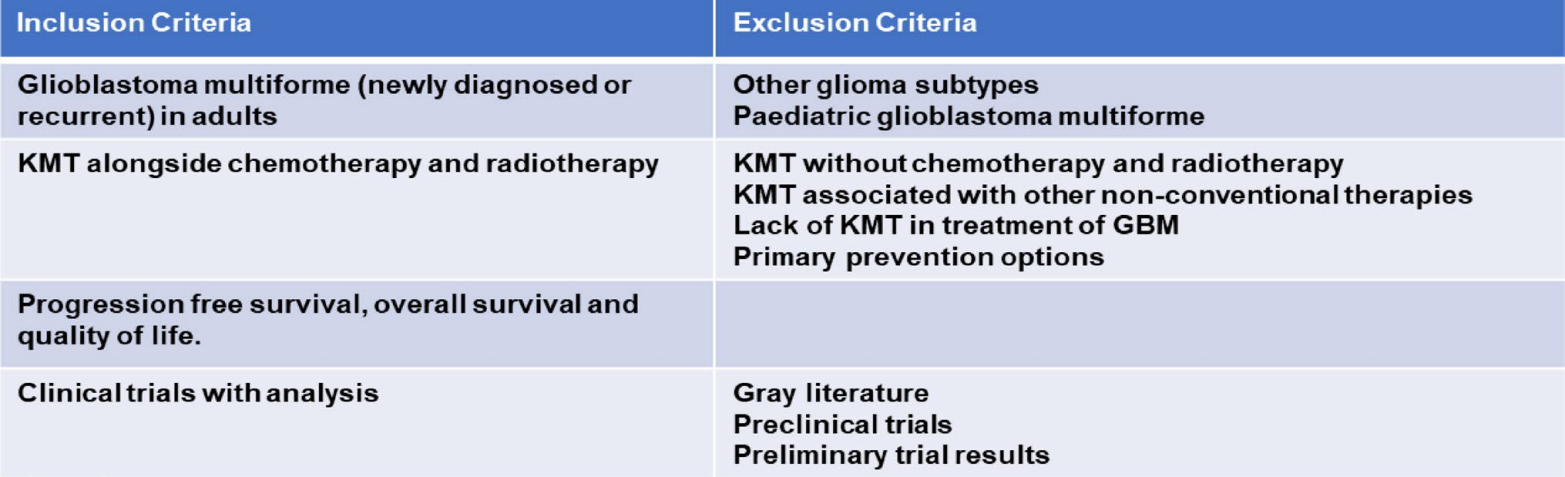
Inclusion and exclusion criteria used to refine the literature found when searching the three databases

**Fig. 1:**
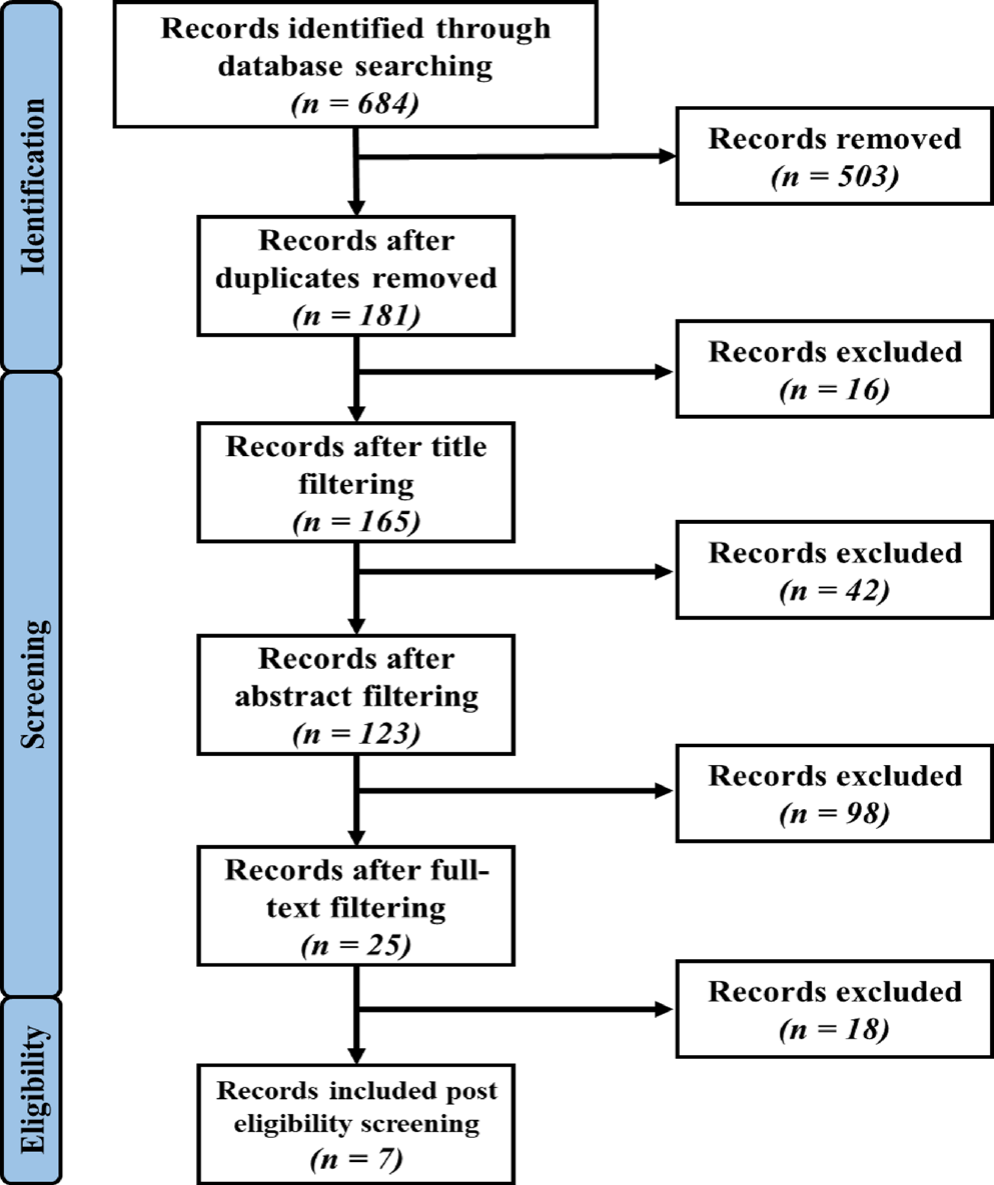
PRISMA Diagram indicating the study selection process

Of the seven papers identified in the selection process (Fig. 1), data was extracted regarding the characteristics of both the study and the participants. The type of study design, presence of control groups and trial objectives were documented. This included composition of KMT, specifically ratios or restrictions and duration. Specific patient characteristics were also recorded, including sample size, average age, tumour stage and previous interventions. Data, if available, on mutation status and tumour location were also included. Studies were excluded if data was lacking on survival outcome measures. The specific outcome measures analysed in the studies include the PFS, OS, incidence of adverse events and side effects.

## Results

### Study characteristics

Seven papers met the criteria following the literature search (Table 2), based between 2014 and 2022, with the most recent in 2022. Only two papers [39]; [40] studied recurrent glioblastoma, with others studying primary glioblastoma. One trial was conducted retrospectively [41], with six prospective, open-label, non-controlled trials. Both Rieger et al. (2014) [39] and Schwartz et al. (2022) [42] used single-arm trials, whereas the KEATING (ketogenic diets as adjuvant therapy for glioblastoma) trial conducted by Martin-McGill et al. (2020) [43], used randomisation to allocate the two possible KD to 12 participants.

**Table 2:**
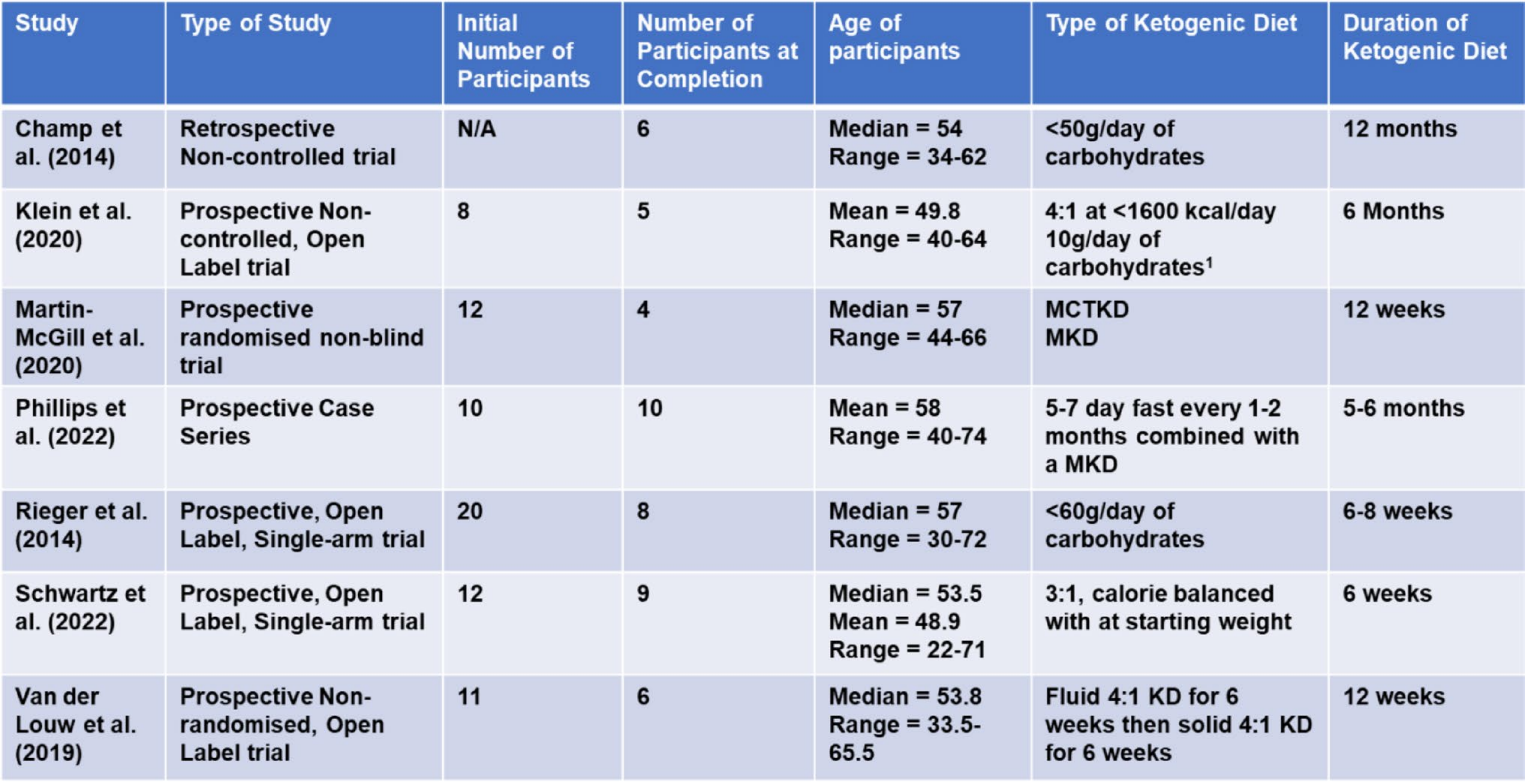
Summary table of study characteristics showing the initial number of patients starting the trial and the number completing. KD = ketogenic Diet; medium chain triglyceride ketogenic Diet = MCTKD; modified ketogenic Diet = MKD. ^1^ if this is not tolerated then the patient would be converted to the 3:1 ratio KD with 20 g/day of carbohydrates

The primary outcome of each study differed: five prospective studies focussed on larger-scale trial feasibility and participant retention rate, while others focussed on the benefit to the patient and the impact on their quality of life. Despite different aims, each trial produced data about patient survival, either as overall (OS) or progression-free survival (PFS).

### Participant characteristics

Each study recruited small cohorts of patients. The average number of patients who began each trial was 11.2 (range of 6 to 20), with an average of 6.9 patients completing the trial (range of 4 to 10). Across the seven studies, the average age varied from 49.8 to 58 years, with the youngest participant aged 22 and the oldest, 74.

Most studies focussed on primary glioblastoma patients, although some included patients with recurrent or secondary glioblastoma. Klein et al. (2020) [40] divided patients into two study groups, one containing four patients with primary glioblastoma and another containing two with secondary glioblastoma and two with recurrent glioblastoma. The ERGO trial [39] did not state tumour stage but detailed the patient’s specific previous treatments. The KEATING trial recruited patients newly diagnosed with glioblastoma and provided patient demographics, including tumour location and mutation status, although the survival data provided did not distinguish between each. The status of both IDH and MGMT mutations were provided by van der Louw et al. (2019) [44]. All nine patients who commenced the KD in this trial were negative for the IDH-1 mutation, conveying better prognoses. Limited data on specific tumour characteristics were provided by Schwartz et al. (2022) [42], with evidence of mutational status lacking. Additionally, the case series by Phillips et al. (2022) [45] showed heterogeneity in the standard treatment regimen. Three participants were diagnosed with inoperable tumours and one patient declined the use of TMZ and chose to be managed palliatively. The lengths of TMZ treatment varied in this study, with only four patients completing the KD concomitant with TMZ therapy.

### Composition of KMT

KMT was initiated for different lengths of time in each study, with the longest lasting 12 months and the shortest of six weeks. Furthermore, each diet consisted of a differing ratio of fats to carbohydrates and protein, and some considered the use of calorie restriction, while others utilised the patient’s body weight to determine baseline calories.

Two studies used a 4:1 KD ratio. In 2019, van der Louw et al. (2019) [44] provided participants with an “exclusively fluid” diet until a ketone level of >3mmol/l was achieved for three consecutive days. Following this, a single 4:1 KD snack was provided. Six weeks post-chemoradiation, a solid-food KD was initiated at a ratio of 1.5–2.0:1, continuing for a further six weeks. Klein et al. (2020) [40] also used this ratio but provided total meal replacements for participants restricting calories to 1600 per day. This cohort was separated into two groups: group 1, where KMT was initiated alongside chemoradiotherapy, and group 2, which initiated KMT post-recurrence, with four subjects in each. Of the cohort, only five completed the six months of KMT, with withdrawals due to disease progression (2) and diet restrictiveness (1).

Klein et al. (2020) [40] implemented a calorie restriction of 1600 kcal for all participants, regardless of age, weight, or gender. This trial focussed on feasibility compared to Schwartz et al. (2022) [42] who focussed on side effects and tumour response. Schwartz et al. (2022) [42] also used a calorie restriction but balanced to the patient’s initial body weight in a 3:1 ratio.

Rieger et al. (2014) [39], supplied participants with 500mls per day of a highly fermented yoghurt drink and two plant oils while restricting a carbohydrate intake to 60 g per day. There was an initial follow-up period of 6–8 weeks where an MRI evaluated tumour progression. If detected, the individual commenced salvage therapy while continuing the diet for another 6–8 weeks.

A modified KD (MKD), with macronutrients containing a minimum of 60% fat, was used by Phillips et al. (2022) [45]. Patients were advised to fast for 5–7 days every 1–2 months. Seven patients continued this diet until it was no longer feasible, usually a month prior to death.

Knowledge of the specific components of the KD used in the retrospective trial is limited. However, of the 134 patients treated for glioblastoma using surgery and chemoradiation, only six adhered to a KD consisting of 77% of calories from fat [41].

Only the KEATING trial compared different KD compositions. 12 patients were recruited and randomised between an MKD and a medium-chain triglyceride ketogenic diet (MCTKD), with six adhering to each [43]. At the primary endpoint of three months, three patients completed the MCTKD, and only one completed the MKD. These four patients continued the diet to the secondary endpoint of 12 months, with only one withdrawing from the MCTKD due to gastrointestinal intolerance.

### Survival outcomes

Each trial measured survival outcomes at differing origin points, Table 3.

**Table 3:**
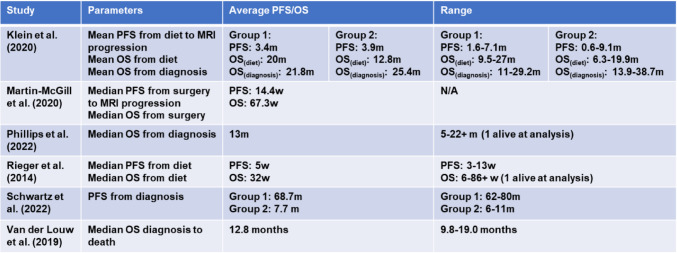
Table showing the overall survival (OS) and progression-free survival (PFS) from the six prospective trials. The data included States the point at which the outcomes are measured from and until. m = month, w = week

Survival was measured at the time of diet initiation by Klein et al. (2020) [40] with results provided about seven of the eight participants; one patient was still alive at the time of analysis and continued the diet independently. From diet initiation until death, there was a mean OS of 20 months in group 1 and 12.8 months in group 2. However, from diagnosis, the mean OS was 21.8 months in group 1 compared to 25.4 months in group 2. Analysis showed two subjects in group 2 developed secondary glioblastoma tumours from grade 3 astrocytomas, contributing to the unexpectedly longer survival time.

The KEATING trial [43] measured survival from the date of surgical intervention. The median OS was 67.3 weeks, compared to the median PFS of 14.4 weeks. Additionally, this trial included a qualitative component analysing patient recruitment and questioning how this could be improved. However, van der Louw et al. (2019) [44] provided data on the median OS outcome measured from the date of diagnosis until death, with results stating an OS of 12.8 months (9.8–19.0 months). Rieger et al. (2014) [39] measured survival from diet initiation; the median OS was 32 weeks (range of 6–86 + weeks). Subsequently, there was a median time to progression, from diet commencement, of five weeks (range of 3–13 weeks).

In the case series by Phillips et al. (2022) [45], seven patients continued the diet until no longer feasible. The median OS was 13 months, with one patient alive (33 + months) at analysis. This compares to Schwartz et al. (2022) [42], where nine participants completed six weeks of the protocol reporting an increased survival in younger patients compared to older counterparts. The three younger patients (aged 32, 28, and 22) had a median PFS from diagnosis of 64 months compared to 7.7 months in the six older patients (mean age of 55 years).

At the time of analysis in the retrospective study, after a follow-up period of 14 months, four of six subjects were alive, with a PFS of 10.3 months [41]. PFS can be impacted by the diagnosis as one deceased patient had a multifocal glioblastoma which is associated with a significantly worse survival than primary glioblastoma [46].

## Discussion

Seven studies were included in this review; for five, the main aim was larger-scale trial feasibility. Only the prospective trial by Schwartz et al. (2022) [42] and the retrospective trial considered the influence on tumour progression and side effects as primary outcomes. Of trials focussed on feasibility, only one used a precise definition, with others merely stating their conclusions. van der Louw et al. (2019) [44] defined feasibility as “at least 60% of patients successfully following the KD for 14 weeks.” 11 began this trial, with two withdrawing immediately. Of the nine initiating the diet, six completed this, equating to 67% retention, meeting the threshold. Therefore, larger trials were deemed possible. In contrast, in the ERGO trial, only 40% of an initial 20 participants reached the endpoint. Despite this reduced retention, conclusions indicated the feasibility of larger trials and suggested further trials considered the effects of calorie restrictions or combination therapies [39]. Other trials provided similar conclusions, with no definitive threshold set but the feasibility of larger clinical trials was stated as possible, these trials will facilitate clear conclusions on the efficacy of KMT with enhanced representation of the general population of glioblastoma patients.

Direct comparisons were limited by heterogeneity between trials. Each trial differed via definitions and measures of ketosis, KD composition and participant characteristics.

Characteristics of participants and tumours varied vastly despite the small sample sizes used. Tumour location and stage, mutation status and treatment regimens differed. Some trials standardised medications between participants through exclusion criteria or adjustments. Specifically, there was incongruent use of dexamethasone between trials. Klein et al. (2020) [40] tapered the dosage of the participants receiving steroid therapy, although two restarted therapy during the trial at the advisement of their oncologists, whereas dexamethasone use was excluded from the van der Louw et al. (2019) [44] trial. However, the KEATING trial did not alter dexamethasone, with varying dosages used and one patient not receiving steroids entirely. Comparing the survival data of these trials can result in discrepancies as steroids influence KMT efficacy through hyperglycaemia. Hyperglycaemia negatively affects survival by providing additional substrate for aerobic glycolysis, mediating tumour growth and increasing the infection rate by reducing neutrophil activity; this is further enhanced by the prescription of steroids [47]. Tumour mutation status also affects comparisons. Mutations affect prognosis, with mutations in the IDH gene conveying favourable outcomes [48] and methylation of MGMT improving prognosis by increasing the response to TMZ [17]. Direct comparisons between these patients will affect the validity of results as the initial prognosis of these patients will differ prior to KD implementation. Some trials considered this when communicating the results, with Schwartz et al. (2022) [42] stating that two IDH mutation-positive patients showed longer survival. Other studies included mutation status, although no reference was made in the results or discussion. Incorrect conclusions may be made about KMT efficacy if these differences are not accounted for when proposing a study. Future research could include subsets within the trials to study the efficacy of KMT in patients with different genetic mutations to directly compare the prognosis for their specific tumour characteristics and understand if the addition of KMT improves survival.

Glioblastoma prevalence increases with age. Those aged 65 or older are 2.63 times more likely to develop glioblastoma than younger patients [49]. Consequently, trials using larger numbers of younger patients do not accurately represent the population of glioblastoma patients. Conclusions by Schwartz et al. (2022) [42] support the higher survival rate of younger patients, as three long-term survivors, aged 32, 28, and 22, were alive at analysis, compared to participants with a mean age of 55, who succumbed to disease progression. Results conveyed that older patients with confirmed ketosis did not benefit from KMT, with suggestions made that future trials focus on younger patients. This compares to the retrospective study (median age of 54 years), which showed reduced serum glucose levels resulting from the KD improved outcomes via anti-angiogenic and anti-tumour effects [41]. Disparities in average ages may suggest the results are not representative, and thus conclusions may not translate to clinical decision-making. Larger clinical trials are necessary to formulate accurate efficacy conclusions representative of the glioblastoma patient population, accounting for differing prognosis-defining characteristics.

The definitions of key parameters differed between studies. Some studies considered OS from the date of diagnosis, including those by van der Louw et al. (2019) [44] and Schwartz et al. (2022) [42], whereas the ERGO trial measured OS and PFS from the date of diet initiation. Data was provided using both starting points by Klein et al. (2020) [40], compared to the KEATING trial, which provided survival from the date of surgical intervention. The differing parameters prevented direct comparisons between trials. Discrepancies are enhanced by conflicting follow-up periods. One study completed an initial 6-week assessment [39], whereas another analysed outcomes at 12 weeks, repeating at 12 months [43]. Moreover, how ketosis was measured differed between trials, with Rieger et al. (2014) [39] defining ketosis through urine ketone. Whereas Phillips et al. (2022) [45] used the therapeutic glucose ketone index (GKI), a ratio of blood glucose to ketone concentration, allowing for standardisation between patients. The lack of standardisation between studies prevented direct comparisons and thus, statistical analysis. Accurate conclusions cannot be made using statistics due to sampling bias and confounding variables. Larger clinical trials indicated by feasibility studies will benefit from standardised measurements, such as GKI, and using the same reference point for survival data. This allows for direct comparison between trials to study the optimum ketogenic ratio and enhance reproducibility.

KD composition differed between each study. Two trials used a carbohydrate restriction, trials by Champ et al. (2014) [41] and Rieger et al. (2014) [39], while others utilised a ratio of fats to carbohydrates, commonly 3:1 or 4:1. Total meal replacement was used by Klein et al. (2020) [40] which allowed for diet standardisation but did not consider individual preferences and requirements. In comparison, Schwartz et al. (2022) [42] implemented a 3:1 KD with calorie restrictions based on the patient’s initial body weight. Due to the heterogeneity in both patient characteristics and parameters, it is not possible to directly compare each KD to ascertain the optimum composition beneficial to patient survival. Standardisation between clinical trials using larger cohorts could provide results detailing the most efficacious KD composition to improve survival in glioblastoma patients.

Trial recruitment was low, with each prospective trial, excluding Phillips et al. (2022) [45] experiencing withdrawal due to diet restrictiveness. Klein et al. (2020) [40] screened an additional 27 patients to the active trial participants, with eight declining due to constraints Moreover, it is easier to monitor compliance with pharmaceutical interventions over dietary interventions as more are likely to adhere due to simplicity and near-immediate effect. Dietary interventions require lifestyle changes and possible external involvement, in some cases, circumstances may not allow for these changes to be feasible.

KMT requires participants to self-certify to evidence compliance. This can result in response bias where participants provide inaccurate results aligning with the trial’s hypothesis as they believe this is wanted. Furthermore, expectancy bias can arise as many patients volunteer for these trials, believing it would work, which can skew data in favour of the intervention [50]. The influence of expectancy bias was discussed in the qualitative component of the KEATING trial, as many patients chose to participate following treatment resistance or tumour recurrence [43]. The inclusion of control groups would enable comparisons of KMT’s efficacy while considering the impact of expectancy bias. Prospective trials included in this review were non-controlled, open-label trials, which prevented the comparison of possible benefits and efficacy of KMT to the general population. Comparing trial data to the OS of the general population of glioblastoma patients will not consider confounding factors including age and compliance. Future clinical trials conducted to study KMT efficacy could include control groups to directly compare the effects of the intervention and minimise potential bias. The unblinded nature of these trials results in selection bias, although specific trials implemented strategies to minimise this. Phillips et al. (2022) [45] completed a case series which can be influenced by selection bias; however, all patients referred were accepted, mitigating this. Contrarily, van der Louw et al. (2019) [44] may have been subject to selection bias as patients requiring dexamethasone were excluded. Other trials [41]; [45] included patients requiring dexamethasone as standard, affecting comparability between trials due to the impact on patient survival.

There was limited understanding of compliance between each trial. Many did not consider the factors which may affect patients’ adherence and thus trial results. Several studies indicated caloric restriction enhanced the metabolic effects of KMT by promoting ketosis and reducing the circulating glucose levels; however, there were large discrepancies in the burden of caloric restriction. Additionally, the burden of caloric restriction should be included in future research to consider the effects of restriction on disease-related symptoms, such as cancer-related cachexia, and the complexity of meal preparation or nutritional counselling.

Research studying KMT and glioblastoma is limited, with only seven out of 165 possible papers meeting the inclusion criteria for this review. This is comparable to the breadth of research focussing on TMZ and glioblastoma. Literature searches of PubMed, following import into EndNote, produced 5,888 papers relevant to TMZ and glioblastoma, with the earliest published in 1994. Conversely, the first published paper for KMT and glioblastoma was a pre-clinical study by Zhou et al. (2007) [51], studying the benefits of a calorie-restricted KD. Conclusions indicated the diet led to anti-tumour and anti-angiogenic effects, which indicated KD’s potential as an alternative treatment. Preclinical trials have provided supportive evidence for the use of KMT, however, the limited evidence in clinical trials prevents sufficient conclusions from being made due to the higher risk of sampling error, as only seven papers were included. Large-scale clinical trials are required to evaluate the effects on quality of life and prognosis, as these trials will be more representative of the general population and provide more accurate results.

Small sample sizes were used in the included trials. An average of 11.2 participants were used with the highest beginning at 20, and only eight reaching the trial’s defined endpoint [39]. Small populations limited conclusions on the statistical impact of KMT on OS. Moreover, small samples produce large standard errors, reducing result validity as standard error determines the result accuracy of sample populations; therefore, the larger the standard error, the less representative trial results are [52]. Larger samples reduce standard error and sampling inaccuracies, increasing the accuracy of statistical tests, and producing more representative results allowing translation to clinical practice.

Limitations of this review include the lack of meta-analysis resulting from trial heterogeneity which prevented direct comparisons between efficacy data. Furthermore, the limited evidence collated during the literature search limited the ability to compose definitive conclusions, as more primary research is required.

This review included secondary data from literature published in major scientific databases, of which each clinical trial received individual ethical approval. As secondary data was used, ethical approval was not required. Despite this, ethical consideration was taken to mitigate bias, prevent plagiarism and data falsification. Screening criteria used was produced prior to the search, and the ethics and conflicts-of-interest statements of each study were critiqued. Conclusions included can enhance understandings of KMT in cancer treatment, providing an insight into the benefits of how dietary changes can influence disease progression. However, this review indicates the need for future research using larger-scale trials to evaluate KMT’s effectiveness using standardised parameters and comparable characteristics.

## Conclusion

Despite extensive literature searching, definitive conclusions concerning KMT’s efficacy in the treatment of glioblastoma cannot be determined. While the clinical trials demonstrated the safety and feasibility of KMT, data was limited surrounding the measures indicating efficacy, and heterogeneity between trials prevented direct comparisons. As a result, accurate conclusions cannot be constructed from the studies included in this review. Moreover, the included prospective trials lacked control groups to compare KD to SD.

The breadth of research conducted on KMT is limited, with few completed clinical trials in comparison to other treatment options for glioblastoma. In addition, the clinical trials completed recruited small numbers of participants, focussing on the feasibility of larger-scale trials, increasing the influence of possible biases on the results. The inconclusive nature of the results in this review highlights the need for more extensive research in this field. Definitive conclusions concerning the efficacy of KMT will require clinical trials involving large cohorts with comparative statistics to the consumption of SD. Study designs should include standardised definitions of specific parameters, including survival outcomes and ketosis, with defined population characteristics to understand the effects on specific classes of glioblastoma.

## Data Availability

No datasets were generated or analysed during the current study.

## Abbreviations

5-ALA: 5-aminolevulinic acid hydrochloride
ATP: Adenosine Triphosphate
GKI: Glucose Ketone Index
IDH: Isocitrate Dehydrogenase Enzymes
KD: Ketogenic Diet
KEATING: Ketogenic Diets as Adjuvant Therapy for Glioblastoma
KMT: Ketogenic Metabolic Therapy
MCTKD: Medium Chain Triglyceride Ketogenic Diet
MGMT: O^6^-methylguanine-DNA methyltransferase
MKD: Modified Ketogenic Diet
MRI: Magnetic Resonance Imaging
OS: Overall Survival
PFS: Progression-free Survival Rate
PRISMA: Preferred Reporting Items for Systematic Reviews and Metanalyses
SD: Standard Diet
TMZ: Temozolomide
TTF: Tumour Treating Fields

## References

[1] Louis DN, Perry A, Wesseling P, Brat DJ, Cree IA, Figarella-Branger D, et al. The 2021 WHO classification of tumors of the central nervous system: a summary. Neuro Oncol. 2021. 10.1093/neuonc/noab106. 34185076 10.1093/neuonc/noab106 PMC8328013

[2] Tran B, Rosenthal MA. Survival comparison between glioblastoma multiforme and other incurable cancers. J Clin Neurosci. 2010;17(4):417–21. 20167494 10.1016/j.jocn.2009.09.004

[3] Tran T, Bruce JN. 2023. Glioblastoma. *NORD (National Organization for Rare Disorders)*. [Online]. [Accessed 24 March 2023]. Available from: https://rarediseases.org/rare-diseases/glioblastoma-multiforme/

[4] Tejaswi Kanderi and Vikas Gupta. 2022. Glioblastoma Multiforme *In*: *StatPearls* [Online]. StatPearls Publishing LLC. [Accessed 24 March 2023]. Available from: https://www.ncbi.nlm.nih.gov/books/NBK558954/32644380

[5] Brain Tumour Charity. 2019. Side Effects. *The Brain Tumour Charity*. [Online]. [Accessed 26 March 2023]. Available from: https://www.thebraintumourcharity.org/living-with-a-brain-tumour/side-effects/

[6] Wirsching H-G, Galanis E, Weller M. Chapter 23 - Glioblastoma M. S. Berger & M. Weller, eds. Handbook of Clinical Neurology. 2016;134:381–397. 10.1016/B978-0-12-802997-8.00023-2 26948367

[7] Guillard F, Sharma R, Rasuli B. 2023. Glioblastoma, IDH-wildtype. Radiopaedia. [Online]. [Accessed 26 March 2023]. Available from: https://radiopaedia.org/articles/glioblastoma-idh-wildtype?lang=us

[8] Urbańska K, Sokołowska J, Szmidt M, Sysa P. Review glioblastoma multiforme – an overview. Wspolczesna Onkol. 2014;18(5):307–12. 10.5114/wo.2014.40559 PMC4248049 25477751

[9] Wu W, Klockow JL, Zhang M, Lafortune F, Chang E, Jin L, et al. Glioblastoma Multiforme (GBM): an overview of current therapies and mechanisms of resistance. Pharmacol Res. 2021;171:105780. 34302977 10.1016/j.phrs.2021.105780 PMC8384724

[10] Hadjipanayis CG, Stummer W. 5-ALA and FDA approval for glioma surgery. J Neurooncol. 2019;141(3):479–86. 30644008 10.1007/s11060-019-03098-y PMC6445645

[11] Weller M, van den Bent M, Hopkins K, Tonn JC, Stupp R, Falini A, et al. EANO guideline for the diagnosis and treatment of anaplastic gliomas and glioblastoma. The Lancet Oncology. 2014;15(9):e395–403. 25079102 10.1016/S1470-2045(14)70011-7

[12] Stupp R, Mason WP, van den Bent MJ, Weller M, Fisher B, Taphoorn MJB, et al. Radiotherapy plus concomitant and adjuvant temozolomide for glioblastoma. N Engl J Med. 2005;352(10):987–96. 15758009 10.1056/NEJMoa043330

[13] Cantidio FS, Gil GOB, Queiroz IN, Regalin M. Glioblastoma — treatment and obstacles. Rep Pract Oncol Radiother. 2022;27(4):744–53. 36196416 10.5603/RPOR.a2022.0076 PMC9521695

[14] Omuro A, DeAngelis LM. Glioblastoma and other malignant gliomas- a clinical review. JAMA. 2013;310(17):1842–50. 24193082 10.1001/jama.2013.280319

[15] Yao M, Li S, Wu X, Diao S, Zhang G, He H, Bian L, Lu Y. Cellular origin of glioblastoma and its implication in precision therapy. Cell Mol Immunol. 2018;15(8):737–9. 29553137 10.1038/cmi.2017.159 PMC6141605

[16] Campos B, Olsen LR, Urup T, Poulsen HS. A comprehensive profile of recurrent glioblastoma. Oncogene. 2016;35(45):5819–25. 27041580 10.1038/onc.2016.85

[17] Thon N, Kreth S, Kreth F-W. Personalized treatment strategies in glioblastoma: MGMT promoter methylation status. Oncotargets Ther. 2013;6:1363–72. 10.2147/OTT.S50208 PMC3792931 24109190

[18] Yan H, Parsons DW, Jin G, McLendon R, Rasheed BA, Yuan W, et al. *IDH1* and *IDH2* mutations in gliomas. N Engl J Med. 2009;360(8):765–73. 19228619 10.1056/NEJMoa0808710 PMC2820383

[19] Wen PY, Reardon DA, Armstrong TS, Phuphanich S, Aiken RD, Landolfi JC, et al. A randomized double-blind placebo-controlled phase II trial of dendritic cell vaccine ICT-107 in newly diagnosed patients with glioblastoma. Clin Cancer Res. 2019;25(19):5799–807. 31320597 10.1158/1078-0432.CCR-19-0261 PMC8132111

[20] Stupp R, Taillibert S, Kanner A, Read W, Steinberg DM, Lhermitte B, et al. Effect of tumor-treating fields plus maintenance Temozolomide vs maintenance Temozolomide alone on survival in patients with glioblastoma. JAMA. 2017;318(23):2306. 29260225 10.1001/jama.2017.18718 PMC5820703

[21] Seyfried TN, Kiebish MA, Marsh J, Shelton LM, Huysentruyt LC, Mukherjee P. Metabolic management of brain cancer. Biochim Et Biophys Acta (BBA) - Bioenergetics. 2011;1807(6):577–94. 20804725 10.1016/j.bbabio.2010.08.009

[22] Zuccoli G, Marcello N, Pisanello A, Servadei F, Vaccaro S, Mukherjee P, et al. Metabolic management of glioblastoma multiforme using standard therapy together with a restricted ketogenic diet: case report. Nutr Metab. 2010;7(1):33. 10.1186/1743-7075-7-33 PMC2874558 20412570

[23] Allen BG, Bhatia SK, Anderson CM, Eichenberger-Gilmore JM, Sibenaller ZA, Mapuskar KA, et al. Ketogenic diets as an adjuvant cancer therapy: history and potential mechanism. Redox Biol. 2014;2:963–70. 25460731 10.1016/j.redox.2014.08.002 PMC4215472

[24] Vidali S, Aminzadeh S, Lambert B, Rutherford T, Sperl W, Kofler B, et al. Mitochondria: the ketogenic diet—a metabolism-based therapy. Int J Biochem Cell Biol. 2015;63:55–9. 25666556 10.1016/j.biocel.2015.01.022

[25] Neal EG, Chaffe H, Schwartz RH, Lawson MS, Edwards N, Fitzsimmons G, Whitney A, Cross JH. The ketogenic diet for the treatment of childhood epilepsy: a randomised controlled trial. Lancet Neurol. 2008;7(6):500–6. 18456557 10.1016/S1474-4422(08)70092-9

[26] Hussain TA, Mathew TC, Dashti AA, Asfar S, Al-Zaid N, Dashti HM. Effect of low-calorie versus low-carbohydrate ketogenic diet in type 2 diabetes. Nutrition. 2012;28(10):1016–21. 22673594 10.1016/j.nut.2012.01.016

[27] Seyfried TN, Marsh J, Shelton LM, Huysentruyt LC, Mukherjee P. Is the restricted ketogenic diet a viable alternative to the standard of care for managing malignant brain cancer? Epilepsy Res. 2012;100(3):310–26. 21885251 10.1016/j.eplepsyres.2011.06.017

[28] Weber DD, Aminzadeh-Gohari S, Tulipan J, Catalano L, Feichtinger RG, Kofler B. Ketogenic diet in the treatment of cancer – where do we stand? Mol Metab. 2019;33:102–21. 31399389 10.1016/j.molmet.2019.06.026 PMC7056920

[29] Jelluma N, Yang X, Stokoe D, Evan GI, Dansen TB, Haas-Kogan DA. Glucose withdrawal induces oxidative stress followed by apoptosis in glioblastoma cells but not in normal human astrocytes. Mol Cancer Res. 2006;4(5):319–30. 16687487 10.1158/1541-7786.MCR-05-0061

[30] Zhu H, Bi D, Zhang Y, Kong C, Du J, Wu X, et al. Ketogenic diet for human diseases: the underlying mechanisms and potential for clinical implementations. Signal Transduct Target Ther. 2022. 10.1038/s41392-021-00831-w. 35034957 10.1038/s41392-021-00831-w PMC8761750

[31] Veech RL. The therapeutic implications of ketone bodies: the effects of ketone bodies in pathological conditions: ketosis, ketogenic diet, redox states, insulin resistance, and mitochondrial metabolism. Prostaglandins Leukot Essent Fatty Acids. 2004;70(3):309–19. 14769489 10.1016/j.plefa.2003.09.007

[32] Abdelwahab MG, Fenton KE, Preul MC, Rho JM, Lynch A, Stafford P, Scheck AC. 2012. The Ketogenic Diet Is an Effective Adjuvant to Radiation Therapy for the Treatment of Malignant Glioma P. Canoll, ed. *PLoS ONE*. 7(5), p.e36197. 10.1371/journal.pone.0036197 PMC3341352 22563484

[33] Jemal M, Molla TS, Asmamaw Dejenie T. Ketogenic diets and their therapeutic potential on breast cancer: a systemic review. Cancer Manag Res. 2021;13:9147–55. 34934359 10.2147/CMAR.S339970 PMC8684375

[34] Pangal DJ, Baertsch H, Kellman EM, Cardinal T, Brunswick A, Rutkowski M, et al. Complementary and alternative medicine for the treatment of gliomas: scoping review of clinical studies, patient outcomes, and toxicity profiles. World Neurosurg. 2021;151:e682-92. 33940275 10.1016/j.wneu.2021.04.096

[35] Martin-McGill KJ, Srikandarajah N, Marson AG, Tudur Smith C, Jenkinson MD. The role of ketogenic diets in the therapeutic management of adult and paediatric gliomas: a systematic review. CNS Oncol. 2018;7(2):CNS17. 29658772 10.2217/cns-2017-0030 PMC5977276

[36] Page MJ, McKenzie JE, Bossuyt PM, Boutron I, Hoffmann TC, Mulrow CD, Shamseer L, Tetzlaff JM, Akl EA, Brennan SE, Chou R, Glanville J, Grimshaw JM, Hróbjartsson A, Lalu MM, Li T, Loder EW, Mayo-Wilson E, McDonald S, McGuinness LA. The PRISMA 2020 statement: An updated guideline for reporting systematic reviews. British Medical Journal. 2021;372(71).

[37] Poff AM, Ari C, Seyfried TN, D’Agostino DP. The Ketogenic Diet and Hyperbaric Oxygen Therapy Prolong Survival in Mice with Systemic Metastatic Cancer C.-H. Tang, ed. PLoS ONE. 2013;8(6):e65522. 10.1371/journal.pone.0065522 PMC3673985 23755243

[38] Santos J, Cruz D, Schï¿½nthal WM, Salazar A, Fontes M, Quirico-Santos CA, T. and, Fonseca D, C. Efficacy of a ketogenic diet with concomitant intranasal Perillyl alcohol as a novel strategy for the therapy of recurrent glioblastoma. Oncol Lett. 2017;15(1):1263–70. 29391903 10.3892/ol.2017.7362 PMC5769394

[39] Rieger J, Bähr O, Maurer GD, Hattingen E, Franz K, Brucker D, et al. ERGO: a pilot study of ketogenic diet in recurrent glioblastoma. Int J Oncol. 2014;44(6):1843–52. 24728273 10.3892/ijo.2014.2382 PMC4063533

[40] Klein P, Tyrlikova I, Zuccoli G, Tyrlik A, Maroon JC. Treatment of glioblastoma multiforme with ‘classic’ 4:1 ketogenic diet total meal replacement. Cancer & Metabolism. 2020. 10.1186/s40170-020-00230-9. 10.1186/s40170-020-00230-9 PMC7653752 33292598

[41] Champ CE, Palmer JD, Volek JS, Werner-Wasik M, Andrews DW, Evans JJ, Glass J, Kim L, Shi W. Targeting metabolism with a ketogenic diet during the treatment of glioblastoma multiforme. J Neurooncol. 2014;117(1):125–31. 24442482 10.1007/s11060-014-1362-0

[42] Schwartz KA, Noel M, Nikolai M, Olson LK, Hord NG, Zakem M, et al. Long-term survivals in aggressive primary brain malignancies treated with an adjuvant ketogenic diet. Front Nutr. 2022;9:770796. 35592625 10.3389/fnut.2022.770796 PMC9112915

[43] Martin-McGill KJ, Marson AG, Tudur Smith C, Young B, Mills SJ, Cherry MG, Jenkinson MD. Ketogenic diets as an adjuvant therapy for glioblastoma (KEATING): a randomized, mixed methods, feasibility study. J Neurooncol. 2020;147(1):213–27. 32036576 10.1007/s11060-020-03417-8 PMC7076054

[44] van der Louw EJTM, Olieman JF, van den Bemt PMLA, Bromberg JEC, de Oomen- Hoop E, Neuteboom RF, et al. Ketogenic diet treatment as adjuvant to standard treatment of glioblastoma multiforme: a feasibility and safety study. Ther Adv Med Oncol. 2019;11:1–13. 10.1177/1758835919853958 PMC6589986 31258628

[45] Phillips MCL, Leyden J, McManus EJ, Lowyim DG, Ziad F, Moon BG, HMY N. a. B., Tan A, Thotathil Z, Jameson MB. Feasibility and safety of a combined metabolic strategy in glioblastoma multiforme: A prospective case series. J Oncol. 2022;2022:4496734. 36276276 10.1155/2022/4496734 PMC9586748

[46] Patil CG, Yi A, Elramsisy A, Hu J, Mukherjee D, Irvin DK, Yu JS, Bannykh SI, Black KL, Nuño M. Prognosis of patients with multifocal glioblastoma: a case-control study. J Neurosurg. 2012;117(4):705–11. 22920963 10.3171/2012.7.JNS12147

[47] Turina M, Fry DE, Polk HC. Acute hyperglycemia and the innate immune system: clinical, cellular, and molecular aspects. Crit Care Med. 2005;33(7):1624–33. 16003073 10.1097/01.ccm.0000170106.61978.d8

[48] Alzial G, Renoult O, Paris F, Gratas C, Clavreul A, Pecqueur C. Wild-type isocitrate dehydrogenase under the spotlight in glioblastoma. Oncogene. 2021;41(5):613–21. 34764443 10.1038/s41388-021-02056-1 PMC8799461

[49] Batchelor T, Shih HA. 2021. Management of Glioblastoma in Older Adults. *UpToDate*. [Online]. [Accessed 25 April 2023]. Available from: https://www.uptodate.com/contents/management-of-glioblastoma-in-older-adults

[50] Williams JB, Popp D, Kobak KA, Detke MJ. P-640 - the power of expectation bias. Eur Psychiatry. 2012;27(1):1.22153731

[51] Zhou W, Mukherjee P, Kiebish MA, Markis WT, Mantis JG, Seyfried TN. The calorically restricted ketogenic diet, an effective alternative therapy for malignant brain cancer. Nutrition & Metabolism. 2007;4(5):5. 17313687 10.1186/1743-7075-4-5 PMC1819381

[52] Kenton W. 2022. How Standard Errors Work. *Investopedia*. [Online]. [Accessed 26 April 2023]. Available from: https://www.investopedia.com/terms/s/standard-error.asp

